# Gut irradiation and microbiota status modulate the meningeal immune landscape

**DOI:** 10.1093/discim/kyag019

**Published:** 2026-09-18

**Authors:** Clara H Whiteley, Urszula M Cytlak, Isobelle L Blair, Samuel J Ashman, Helen Parker, Timothy M Illidge, Douglas P Dyer, Mark A Travis, Andrew D Greenhalgh

**Affiliations:** Lydia Becker Institute of Immunology and Inflammation, Faculty of Biology, Medicine and Health, Manchester Academic Health Science Centre, The University of Manchester, Manchester, UK; Geoffrey Jefferson Brain Research Centre, Manchester Academic Health Science Centre, Northern Care Alliance NHS Group, The University of Manchester, Manchester, UK; Manchester Centre for Cell-Matrix Research, Faculty of Biology, Medicine and Health, Manchester Academic Health Science Centre, The University of Manchester, Manchester, UK; Lydia Becker Institute of Immunology and Inflammation, Faculty of Biology, Medicine and Health, Manchester Academic Health Science Centre, The University of Manchester, Manchester, UK; Manchester Centre for Cell-Matrix Research, Faculty of Biology, Medicine and Health, Manchester Academic Health Science Centre, The University of Manchester, Manchester, UK; Lydia Becker Institute of Immunology and Inflammation, Faculty of Biology, Medicine and Health, Manchester Academic Health Science Centre, The University of Manchester, Manchester, UK; Manchester Centre for Cell-Matrix Research, Faculty of Biology, Medicine and Health, Manchester Academic Health Science Centre, The University of Manchester, Manchester, UK; Lydia Becker Institute of Immunology and Inflammation, Faculty of Biology, Medicine and Health, Manchester Academic Health Science Centre, The University of Manchester, Manchester, UK; Geoffrey Jefferson Brain Research Centre, Manchester Academic Health Science Centre, Northern Care Alliance NHS Group, The University of Manchester, Manchester, UK; Manchester Centre for Cell-Matrix Research, Faculty of Biology, Medicine and Health, Manchester Academic Health Science Centre, The University of Manchester, Manchester, UK; Lydia Becker Institute of Immunology and Inflammation, Faculty of Biology, Medicine and Health, Manchester Academic Health Science Centre, The University of Manchester, Manchester, UK; Geoffrey Jefferson Brain Research Centre, Manchester Academic Health Science Centre, Northern Care Alliance NHS Group, The University of Manchester, Manchester, UK; Manchester Centre for Cell-Matrix Research, Faculty of Biology, Medicine and Health, Manchester Academic Health Science Centre, The University of Manchester, Manchester, UK; Targeted Therapy Group, Division of Cancer Sciences, School of Medical Sciences, Faculty of Biology, Medicine and Health, The University of Manchester, Manchester, UK; Christie NHS Foundation Trust, Manchester Academic Health Science Centre, Manchester, UK; Lydia Becker Institute of Immunology and Inflammation, Faculty of Biology, Medicine and Health, Manchester Academic Health Science Centre, The University of Manchester, Manchester, UK; Geoffrey Jefferson Brain Research Centre, Manchester Academic Health Science Centre, Northern Care Alliance NHS Group, The University of Manchester, Manchester, UK; Manchester Centre for Cell-Matrix Research, Faculty of Biology, Medicine and Health, Manchester Academic Health Science Centre, The University of Manchester, Manchester, UK; Lydia Becker Institute of Immunology and Inflammation, Faculty of Biology, Medicine and Health, Manchester Academic Health Science Centre, The University of Manchester, Manchester, UK; Manchester Centre for Cell-Matrix Research, Faculty of Biology, Medicine and Health, Manchester Academic Health Science Centre, The University of Manchester, Manchester, UK; Lydia Becker Institute of Immunology and Inflammation, Faculty of Biology, Medicine and Health, Manchester Academic Health Science Centre, The University of Manchester, Manchester, UK; Geoffrey Jefferson Brain Research Centre, Manchester Academic Health Science Centre, Northern Care Alliance NHS Group, The University of Manchester, Manchester, UK; Manchester Centre for Cell-Matrix Research, Faculty of Biology, Medicine and Health, Manchester Academic Health Science Centre, The University of Manchester, Manchester, UK

**Keywords:** radiotherapy, gut–brain axis, meninges, immunity

## Abstract

Radiotherapy (RT) is an important part of curative treatment in many common abdominal and pelvic cancers. However, the gastrointestinal toxicities can affect the quality of life and outcomes for many patients. The underlying local gut toxicity and systemic effects, including effects on mental health, are underexplored and poorly understood. Given emerging evidence of immunological communication across the gut–brain axis, we investigated whether abdominal RT could induce distal immune alterations in the dural meninges and explored potential behavioural outcomes. Using a preclinical model of image-guided abdominal RT treatment, we induced a local myeloid-driven inflammatory response in the small intestine and colon. We observed significant systemic immune modulations in the peripheral blood and dural meninges, affecting a diverse range of immune cells. Whilst circulating immune changes largely resolved alongside recovery of intestinal inflammation, dural immune alterations were prolonged and dynamically shifted across time points. Depletion of the gut microbiota partially attenuated both local and systemic immune responses, particularly affecting RT-induced increases in neutrophil and monocyte populations, implicating microbiota-dependent mechanisms in mediating these effects. After recovery from acute intestinal toxicity, RT-treated mice displayed increased anxiety-like phenotypes without evidence of cognitive impairment. Collectively, these findings demonstrate that gut-targeted RT can drive systemic immunomodulatory effects that extend to the brain border tissues in a partially microbiota-dependent manner and can enhance anxiety-like behaviour in mice. This highlights the systemic immune consequences of RT beyond the targeted tissue site, and the potential importance of RT-mediated immune organ crosstalk in mediating therapy outcomes.

## Graphical Abstract

**Graphical Abstract:**
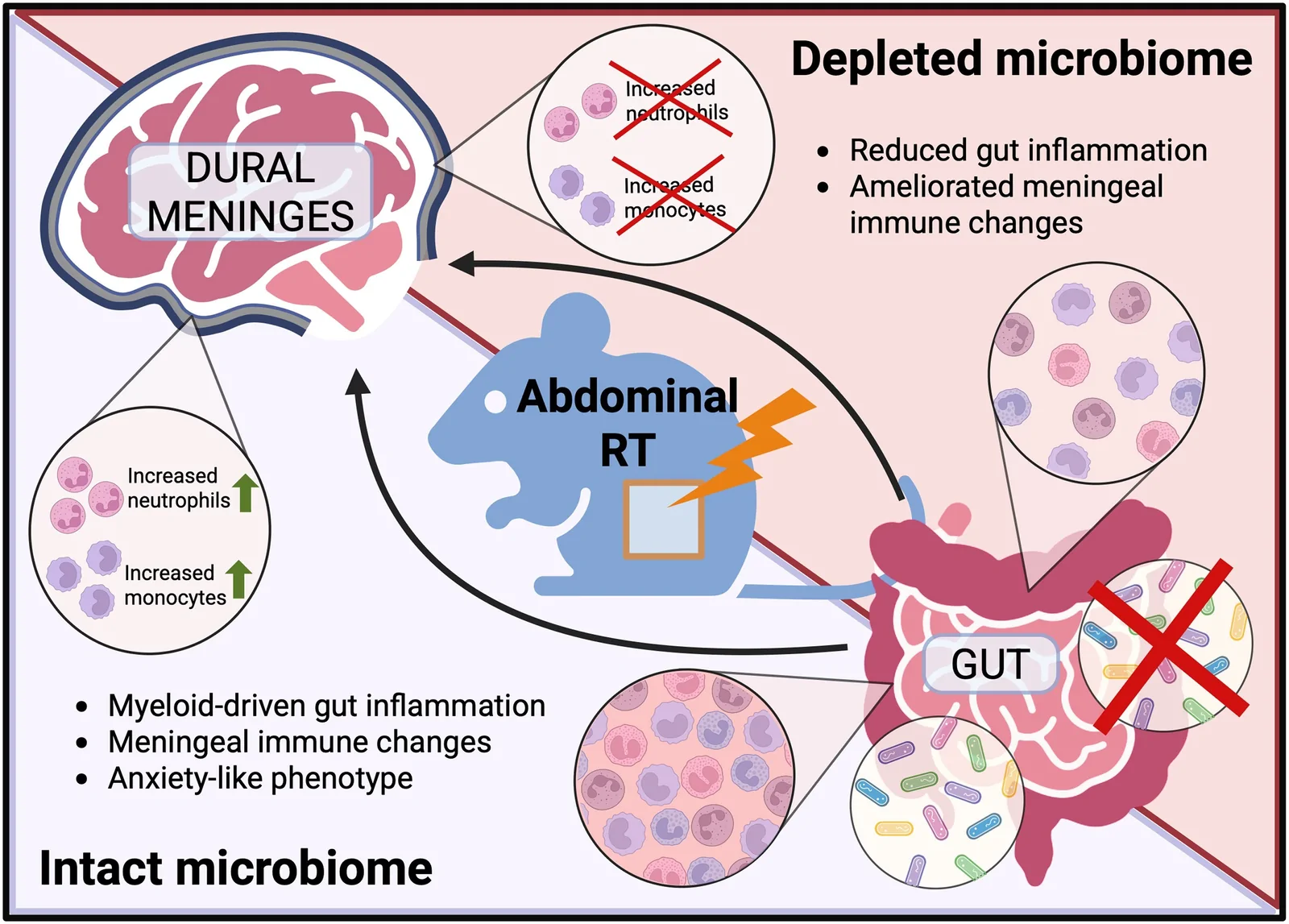

## Introduction

Radiotherapy (RT) is a critical treatment option for many common abdominal and pelvic cancers, including rectal, prostate, gynaecological, bladder, and pancreatic cancers. Despite improvement in the precision of advanced RT delivery, the surrounding normal tissues continue to receive significant RT doses that lead to radiation-induced intestinal injury (RIII). Around two-thirds of patients experience RT-induced acute toxicities, which are associated with symptoms of abdominal pain, nausea and vomiting, diarrhoea, incontinence, bleeding, and mucus discharge, and roughly one-third experience psychological distress including anxiety, depression, and fatigue [1–4]. The underlying mechanisms of RIII remain poorly understood but are often attributed to tissue injury, cell death, loss of gut barrier integrity, and acute inflammation [2, 5]. The psychological consequences of fatigue, anxiety, and depression are considered secondary to the debilitating physical effects of treatment and emotional stress of cancer diagnosis. However, more recently, the molecular mechanisms and biological function of immune organ crosstalk are increasingly recognized, with interactions between the brain, heart, and gut receiving the most attention in organ connection studies [6].

Preclinical models of abdominal RT, alongside patient studies, have allowed a deeper understanding of the cellular pathways involved in RIII and have highlighted roles for the gut microbiota and gastrointestinal (GI) immune system in mediating damage-associated pathologies in manners that can be protective or detrimental in different contexts [7–12]. Gut barrier breakdown, microbiota dysbiosis, and intestinal inflammation can drive systemic inflammatory responses affecting the homeostasis of distal tissues, including the brain. In particular, gut dysbiosis and inflammatory bowel diseases have been strongly associated with increased incidences of anxiety and depression [13, 14]. Abdominal RT is known to induce dysbiosis and inflammation in the GI tract [10, 15, 16], however, studies investigating possible effects of abdominal RT on anxiety and depression, and the perturbations it might have in central nervous system (CNS-)associated distal tissues are lacking.

The meninges surrounding the brain and spinal cord of the CNS are composed of three membrane layers that act as an interface between the CNS and the periphery. The meningeal immune compartment plays an important role in protecting the brain against pathogens whilst also being important for regulating brain homeostasis and shaping cognition and behaviour in mice [17–21]. The outermost meningeal layer, known as the dura mater, is the main immune hub of the meninges and is a highly vascularized tissue. However, unlike the vasculature of the CNS parenchyma, the dural vessels are fenestrated and therefore permissive to immune cell trafficking and the movement of larger molecules entering the dural stroma from the circulation. Peripheral inflammation can influence meningeal immune phenotypes [22], and emerging evidence demonstrates immunological links that exist across the gut–meninges axis whereby the gut microbiota and local inflammatory stimuli in the gut can influence immune reactivity in the dural meninges [23–26]. However, whether and how abdominal RT may influence the meningeal immune landscape is under-investigated and unknown.

Therefore, we investigated whether abdominal RT could induce dural immune cell changes using a preclinical mouse model previously established to explore immune changes in the intestine [10]. Using this model, we demonstrate that RT targeted to the intestine induces systemic immunomodulatory effects in the dural meninges, and that the gut microbiota plays a role in driving these systemic responses. Furthermore, we demonstrated that abdominal RT treatment induced anxiety-like behaviours in mice. Together, our results show that abdominal RT leads to systemic immunomodulatory and functional effects within the CNS, highlighting the need to further understand mechanisms in developing therapeutic interventions to further improve cancer outcomes.

## Materials and methods

### Experimental animals

Female C57BL/6J mice were purchased from Charles River Laboratories and housed in specific pathogen-free conditions under a 12-hour light/dark cycle, with standard laboratory food and water provided *ab libitum*. Mice were between the ages of 10 and 12 weeks at the start of any RT procedures, and following treatment, mice were fed a diet of mash and closely monitored for well-being. All experiments were reviewed by the University of Manchester Animal Welfare and Ethical Review Board and were performed in accordance with the Home Office Scientific Procedures Act (1986).

### SARRP irradiation

RT was delivered using a Small Animal Radiation Research Platform (SARRP; Xstrahl Ltd.). Mice were anaesthetized with isoflurane and placed stomach-down on a stage, receiving continuous anaesthesia via nose cone. The mice received a cone-beam computed tomography (CBCT) scan, from which a 3D reconstructed image was generated and used within MuriPlan Software to define the targeted region and calculate radiation dose. A 10 × 10 mm collimator was used to deliver a total of 13 Gy X-ray radiation (at 220 kV and 13 mA) to the left and right flanks of the mice in two parallel opposed beams, with care taken to avoid the bone marrow and other nearby organs such as the liver and kidneys. Control groups underwent a sham procedure where mice were anaesthetized and CBCT scanned, but no further radiation was delivered.

### Antibiotic treatment

Mice were treated with a broad-spectrum antibiotic cocktail containing ampicillin, neomycin, metronidazole, gentamicin (all at 1 g/L), and vancomycin (0.5 g/L) in their drinking water, with saccharine added to increase palatability, as described previously [27]. Control animals received water with saccharine only, and water was refreshed weekly. Mice were treated with antibiotics for 2 weeks before RT and continuously given antibiotic water until the end of the experiment.

### Sample collection

Blood was collected via cardiac puncture immediately prior to cardiac perfusion with 10 ml ice-cold phosphate-buffered saline (PBS). The small intestine and colon were collected, and the dorsal skull cap was isolated with the brain removed. Using a dissection microscope, the dural meningeal layer was separated from the inner surface of the skull cap using fine forceps, beginning at the tissue margins and carefully peeling the membrane away from the underlying bone.

### CD45 intravenous (IV) labelling

For CD45 IV labelling, mice were injected IV with 200 µl anti-CD45.2 antibody conjugated to fluorescein isothiocyanate (FITC) (BioLegend; 109806) at a concentration of 0.1 µg/ml. Mice were immediately anaesthetized and prepped for cardiac perfusion, with perfusions being performed 3–5 min after injection.

### Tissue processing for histology

Tissue sections of the small intestine and colon were fixed overnight in formalin and transferred to 70% ethanol. Tissue was processed overnight using the Leica ASP 300 tissue processor (Leica Biosystems) and paraffin-embedded. 5 µm sections were transversely cut and stained with haematoxylin and eosin (H&E). Slides were imaged with a Panoramic 250 Digital Scanner (3D Histech).

### Tissue processing for flow cytometry

#### Blood

Blood was collected in an Eppendorf tube coated with 0.5 M ethylenediaminetetraacetic acid (EDTA) (Sigma-Aldrich, 03690) to prevent clotting. Samples were incubated in 4 ml red blood cell lysis buffer (Gibco, A10429–01) for 15 min before being washed with PBS ahead of staining.

#### Small intestine and colon

The small intestine and colon were cleaned and weighed. Tissues were incubated in 3% Roswell Park Memorial Institute (RPMI) media (media described in Table 1) with 1 mM dithiothreitol (DTT) (Sigma-Aldrich, D0632) and 5 mM EDTA (Sigma-Aldrich, 03690) at 37°C under agitation (200 rpm) for 20 min. Tissues were then washed by shaking in a 50 ml Falcon tube in Shake media (Table 1), with media refreshed three times before a final wash step by shaking in PBS. The tissues were then manually chopped using scissors, before being enzymatically digested in complete RPMI media (cRPMI, Table 1) with 0.1 mg/ml Liberase TL (Roche, 05401020001) and 4 mg/ml DNase I (Sigma-Aldrich, DN25) at 37°C under agitation (200 rpm) for 30 min. After incubation, 3% Fetal Bovine Serum (Gibco, A5256801) was added to stop the enzyme reactions, and the tissue was passed through a 40 µm strainer with 3% RPMI media to produce a single-cell suspension.

**Table 1:** Media preparations for tissue processing.

Media	Reagents	Supplier
	RPMI with L-glutamine and sodium bicarbonate	Sigma-Aldrich; R8758
3% RPMI	1% penicillin-streptomycin	Sigma-Aldrich; P0781
	3% fetal bovine serum	Gibco; A5256801
	20 mM 4-(2-hydroxyethyl)-1-piperazineethanesulfonic acid (HEPES)	Sigma-Aldrich; H0887
	RPMI with L-glutamine and sodium bicarbonate	Sigma-Aldrich; R8758
Shake	1% penicillin-streptomycin	Sigma-Aldrich; P0781
	2 mM EDTA	Sigma-Aldrich; 03690
	20 mM HEPES	Sigma-Aldrich; H0887
	RPMI with L-glutamine and sodium bicarbonate	Sigma-Aldrich; R8758
cRPMI	1% penicillin-streptomycin	Sigma-Aldrich; P0781
	1% minimum essential medium (MEM) non-essential amino acid solution	Sigma-Aldrich; M7145
	1% sodium pyruvate 24 mM HEPES	Gibco; 11360070 Sigma-Aldrich; H0887
	1X PBS	Sigma-Aldrich; D8637
FACS buffer	0.5% bovine serum albumin	Sigma-Aldrich; A7906
	2 mM EDTA	Sigma-Aldrich; 03690

#### Dural meninges

The extracted dural meninges were incubated in a digestion medium of Hanks’ Balanced Salt Solution (Sigma-Aldrich, H9269) with 4 U/ml Liberase TL (Roche, 05401020001) and 160 U/ml DNase I (Sigma-Aldrich, D5025) at 37°C under mild agitation (200 rpm) for 30 min. The tissue was dissociated via repetitive trituration with a 1 ml micropipette and passed through a 70 µm cell strainer with fluorescence-activated cell sorting (FACS) buffer to produce a single-cell suspension.

For all tissues, CountBright Absolute Counting Beads (Invitrogen, C36950) were added to the single-cell suspensions before staining. Zombie UV (1:2000; BioLegend, 423108) acted as a live/dead marker, and Fc receptors were blocked with anti-CD16/CD32 antibody (1:100; BD Biosciences, 553142). Cells were stained with directly conjugated monoclonal antibodies (listed in Table 2).

**Table 2:** Conjugated antibodies used for flow cytometry analysis.

Antibody target	Conjugate	Dilution/concentration	Supplier
CD45	BUV805	1:400	BD Biosciences; 748370
CD11b	BUV737	1:1200	BD Biosciences; 612800
F4/80	BUV563	1:400	BD Biosciences; 749284
CD11c	BV785	1:400	BioLegend; 117336
TCRβ	BV711	1:600	BioLegend; 109243
IA-IE (MHCII)	BV650	1:2000	BioLegend; 107641
CD4	BV605	1:600	BioLegend; 100548
XCR1	BV510	1:400	BioLegend; 148218
CD64	BV421	1:200	BioLegend; 139309
Ly6C	BB700	1:1400	BD Biosciences; 755203
CX3CR1	FITC	1:200	BioLegend; 149020
CD163	PE/Cy7	1:800	BioLegend; 155319
γδ	PE-CF594	1:400	BD Biosciences; 563532
Siglec-F	PE	1:200	BD Biosciences; 552126
CD19	APC/Cy7	1:200	BioLegend; 115530
Ly6G	AF700	1:200	BioLegend; 127622
CD8α	APC	1:400	BioLegend; 100712
CD8b.2	APC	1:400	BioLegend; 140410
CD45.2	FITC	0.1 µg/ml	BioLegend; 109806

### Flow cytometry acquisition and analysis

All samples were acquired with the BD FACSymphony (BD Biosciences), using FACSDiva software. The data output was quantified using FlowJo™ Software (version 10.9), and manual gating strategies (Supplementary Figures S1–S3) were used to define immune cell subsets. For the small intestine and colon, counting beads were used to calculate total cell number per gram of tissue. For the blood, counting beads were used to calculate total cell number per millilitre of blood. For the dural meninges, the whole sample was run through the flow cytometer to obtain total cell count per dura. Time course analysis of the meninges and blood is presented as fold-change in immune cell counts, normalized to experimental sham controls.

### Behavioural testing

Following recovery from RT treatment, animals were subjected to behavioural testing including the open field (OF) test, elevated plus maze (EPM) test, and novel object recognition (NOR) test. OF and EPM tests were carried out 14 days post-RT treatment, and NOR was performed 15/16 days post-RT. Animals were acclimatized to the testing room for at least 1 h before the start of any behavioural testing. In between each test, the apparatus was cleaned with 70% ethanol to eliminate odour effects. No more than one behavioural test per mouse was completed during any single day.

### Open field

The OF test was used to analyse exploratory activity and anxiety-like behaviours. The apparatus consisted of a 43 cm by 43 cm open-air box, and mice were introduced to the box the day before testing to allow for acclimatization. For the test, mice were placed in the centre of the box and allowed to explore freely. Their movements were recorded for 10 min, and AnyMaze software (version 7.40) was used to analyse total distance travelled (metres; m) and time spent in a defined inner quadrant (seconds; s).

### Elevated plus maze

The EPM test was used to test for anxiety-like behaviours. The maze consisted of two open arm platforms and two closed arm platforms which extended out from a central area in a ‘plus’ shape. The arms were 80 cm in length, 5 cm in width, and stood 60 cm elevated from the ground. The closed arms were enclosed by 35 cm high black walls. In the test, mice were placed in the centre of the maze and allowed to freely explore. Their movements were video-recorded for 5 min, and AnyMaze software was used to generate data on the number of entries into the open arm zone and time spent in the open arms (s).

### Novel object recognition

The NOR test was used to measure short-term learning and recognition memory. During the first phase of the task, mice were placed in the OF arena described above and left to explore two identical objects for 10 min. Mice were then removed from the box, and one of the objects was swapped out for a novel object. After a 2-hour latency period, mice were put back in the arena and allowed to explore for 6 further minutes. This second exploration was recorded, and time spent investigating the familiar and novel objects was manually recorded. Investigation of an object was defined as sniffing, nose touching, or front paws touching. Climbing on top of the object and chewing were not considered exploratory behaviours. DI was calculated as (time exploring novel object—time exploring familiar object)/(time exploring novel object + time exploring familiar object).

### Statistical analysis

All data sets were tested for normality using the Shapiro–Wilk test. Statistical comparisons were performed using unpaired *t*-tests, one-way ANOVA with Dunnett’s multiple comparisons test, and two-way ANOVA with Fisher’s least significant difference (LSD) post-hoc test, as appropriate. When applicable, non-parametric Mann–Whitney tests, Kruskal–Wallis tests with Dunn’s multiple comparisons test, and aligned rank transform (ART) analyses were used. Groups were considered statistically different when *P* < 0.05. Analyses were performed using GraphPad Prism and R with the ARTool package. Data are presented as mean ± SD.

## Results

### Gut-targeted RT induces a local inflammatory response in the small intestine and colon

To investigate the local and systemic immune effects of RT treatment, mice were treated with a single 13 Gy dose of radiation using a SARRP, image-guided to a 10 × 10 mm region within the abdomen (Fig. 1a). The radiation dose was precisely localized to the small intestine and colon as previously described [10]. Compared to other models that use lead shielding or total body irradiation, this image-guided approach improves the specificity of dose delivery and avoids hitting the bone marrow and other off-target organs [10]. Consequently, any observed systemic effects can be attributed to the highly precise localized radiation-induced intestinal tissue damage rather than off-target radiation effects.

**Figure 1:**
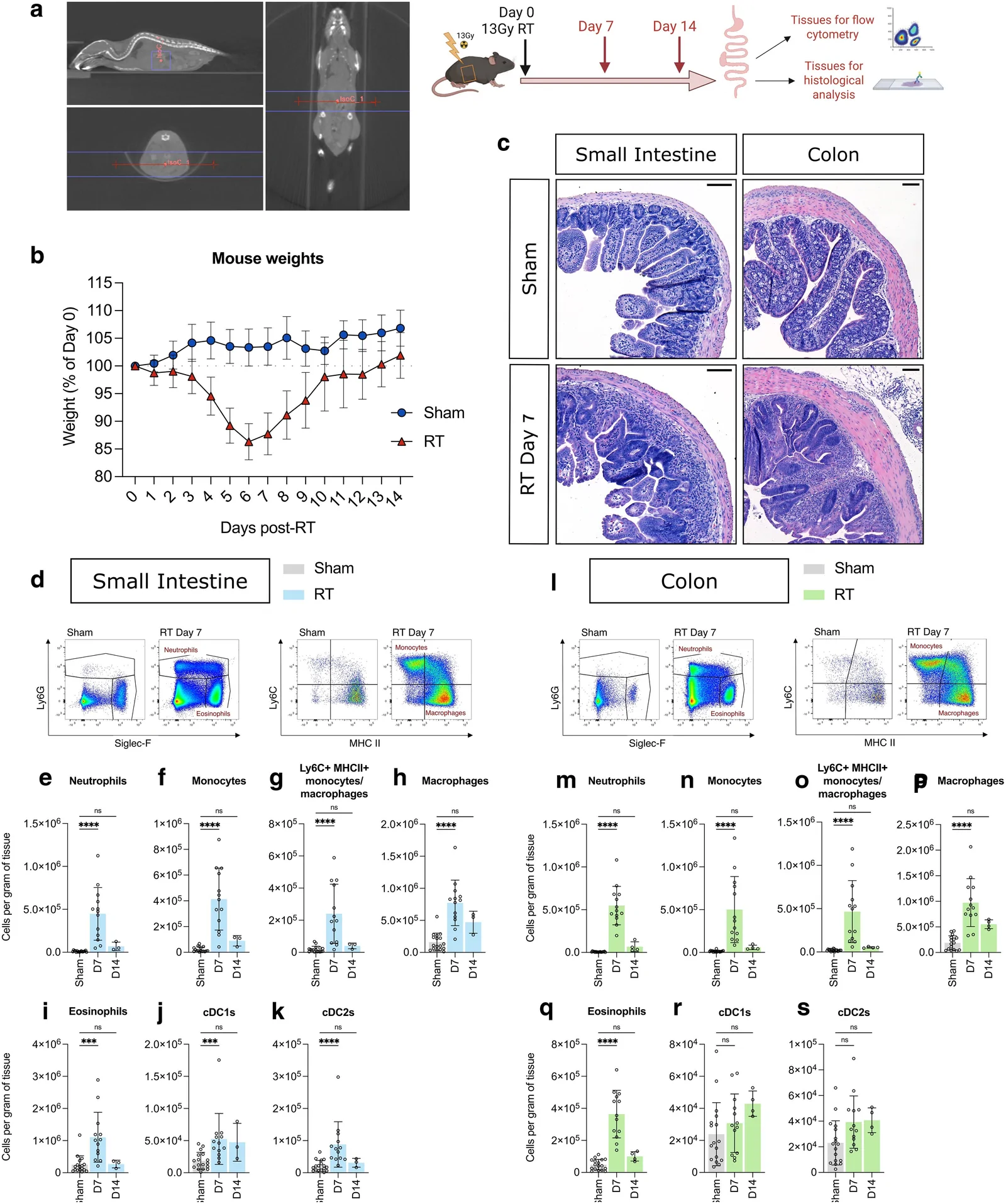
Local inflammatory responses in the small intestine and colon following RT. (a) Image-guided delivery of a 13 Gy dose of X-ray RT targeted to the abdomen. Small intestine and colon tissues were collected at days 7 (D7) and 14 (D14) post-treatment for histological and flow cytometry analysis. (b) Percentage body weight loss in irradiated vs sham mice between days 0 and 14 following treatment. (c) Representative H&E-stained images of the small intestine (left) and colon (right) in sham (top) and irradiated (bottom) animals, at D7 post-treatment (scale bar, 100 µm). (d-s) Flow cytometry time course analysis with representative D7 flow plots (d and l) showing changes in immune cell counts in the small intestine (d-k) and colon (l-s) of sham vs irradiated mice. At least *n* = 3 irradiated mice were included per time point, and shams from each time point were pooled. Data were combined from multiple experiments. (e-k, m-s) Data analysed by Kruskal–Wallis test with Dunn’s post-hoc test. Data are presented as means ± SD. *P < 0.05 ***P < 0.001 ****P < 0.0001.

In agreement with previous data [10], RT treatment induced weight loss that peaked between days 6 and 7 post-RT but resolved to baseline weights by days 13 and 14 (Fig. 1b), therefore we chose to collect tissues at day 7 and day 14 time points to represent peak inflammatory and resolution stages. At day 7, post-RT H&E staining of luminal cross-sections of the small intestine and proximal colon demonstrated tissue damage, loss of stem cell-rich crypt organization, and influx of inflammatory cells (Fig. 1c). Flow cytometry analysis of the small intestine at day 7 post-RT showed substantial inflammation marked by significant increases in myeloid immune cell numbers, specifically neutrophils, recruited monocytes, macrophages, eosinophils, conventional type 1 dendritic cells (cDC1 s), and conventional type 2 dendritic cells (cDC2 s) (Fig. 1d–k; gating strategy in Supplementary Figure S1a). However, the local inflammation within the small intestine did not impact lymphocyte cell numbers, with no differences in B cell, CD4+ T cell, CD8+ T cell, or γδ T cell counts (Supplementary Figure S1b–e). Although only a portion of the distal colon resides within the irradiation field [10], analysis of the colon also exhibited a large inflammatory response at day 7 post-RT, marked by significant expansions in neutrophils, recruited monocytes, macrophages, and eosinophils (Fig. 1l–q). There were no significant differences seen in the cDC1 or cDC2 cell populations (Fig. 1r and s) or in B cell, CD8+ T cell, or γδ T cell numbers in the colon at day 7 post-RT, but we did observe increases in CD4+ T cells (Supplementary Figure S1f–i). In line with recovered body weights, inflammation within the small intestine and colon had largely subsided by day 14 post-RT, with no significant differences in immune cell counts between shams and irradiated animals at this timepoint (Fig. 1d–s). Together, consistent with previous data [10], these results show that irradiation targeted to the small intestine and proximal colon causes local tissue damage associated with a myeloid-driven immune response that is heightened at day 7 but resolves by day 14 post-treatment, whilst intestinal lymphocyte populations remain largely unaffected.

### Gut-targeted RT induces systemic immune changes in the dural meninges

A growing body of evidence indicates that immunological crosstalk occurs between the gut and the meninges, which links intestinal immunity and microbiota status to central nervous system defences [25, 28, 29]. In homeostasis, immune cells with gut origins can be seen in the dural meninges [29], and immune populations within the dura are modulated in response to localized gut inflammatory insults [23, 24]. We therefore wanted to investigate whether the local inflammation that affects the GI tract following RT treatment could impact the brain border tissues of the dural meninges. To test this, mice were treated with 13 Gy abdominal irradiation, and the dura mater was analysed by flow cytometry at days 7, 14/15, and 20/21 post-RT (Fig. 2a; gating strategy, mean cell counts and total CD45+ counts in Supplementary Figure S2a–c). Fold-changes in immune cell counts per dural meninges were calculated and normalized to experimental sham controls.

**Figure 2:**
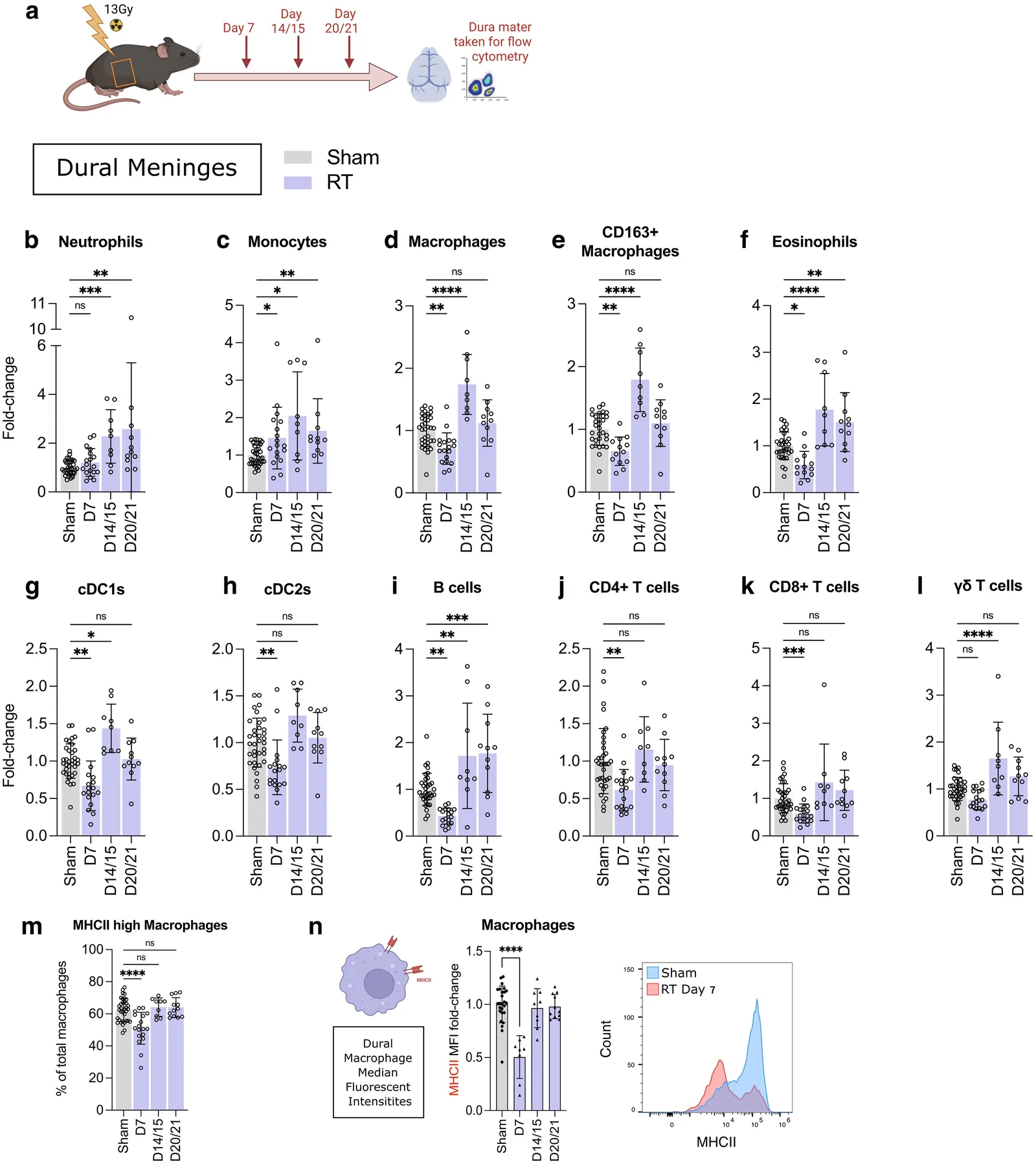
Immune cell changes in the dural meninges following abdominal RT. (a) Mice received 13 Gy abdominal RT, and the dura mater was collected following cardiac perfusion with PBS at day 7 (D7), day 14 or 15 (D14/15), and day 20 or 21 (D20/21) post-treatment. (b-l) Flow cytometry time course analysis of changes in immune cell counts in sham vs irradiated mice. Data are presented as fold-change in total cell counts per dural meninges tissue extracted compared to experimental sham controls. (m) Proportion of MHCII-high-expressing macrophages as a percentage of total macrophages. (n) Expression of MHCII on dural macrophages measured by MFI with representative histogram at D7 timepoint. Data were combined from multiple experiments with shams pooled across each experiment. At least *n* = 9 irradiated mice were included per timepoint. (b-n) Data analysed by One-way ANOVA with Dunnett’s post-hoc test or Kruskal–Wallis test with Dunn’s post-hoc test where appropriate. Data are presented as means ± SD. *P < 0.05 **P < 0.01 ***P < 0.001 ****P < 0.0001.

Interestingly, following gut-targeted RT, there were significant shifts in dural immune cell counts across all subsets investigated. In the myeloid immune compartment, neutrophils were unchanged at day 7 post-RT, but by days 14/15 and 20/21 were significantly increased in cell numbers compared to sham controls (Fig. 2b). Similarly, monocytes were elevated across all time points (Fig. 2c), suggesting sustained signals for recruitment of neutrophils and monocytes to the dural meninges up to at least 3 weeks post-treatment. In contrast, total macrophages, CD163+ border-associated macrophages (BAMs), eosinophils, cDC1 s and cDC2 s had significantly reduced counts at day 7 compared to sham animals (Fig. 2d–h). By day 14/15, once inflammation within the gut was largely resolved, these immune cell types had re-established cell numbers, but to a point that was significantly elevated above sham controls (Fig. 2d–g). These immune cell numbers were restored back to baseline levels by day 20/21, with the exception of eosinophils, which remained elevated (Fig. 2f). Thus, these data suggest a possible overcompensation when restoring meningeal immune cell numbers back to baseline levels following an initial reduction in cell counts during the peak of gut inflammation.

Despite intestinal lymphocyte counts being unchanged during the peak inflammatory state post-RT, B cells, CD4+ T cells, and CD8+ T cells in the dural lymphoid compartment were significantly decreased in number at day 7 following RT (Fig. 2i–k). By day 14/15, B cells and γδ T cells had become significantly elevated compared to shams (Fig. 2i and l). Elevated B cell numbers persisted at day 20/21, whilst γδ T cells returned to normal levels. CD4+ and CD8+ T cells had returned to baseline numbers by day 14/15 (Fig. 2j and k).

Together, this time course analysis demonstrates that the numbers of most dural immune cell types are impacted by RT treatment targeting the GI tract, with monocytes, neutrophils, eosinophils, and B cells showing significant and persistent elevation in number. However, for the B cells and eosinophils, persistent elevation at the later time points occurred following an initial significant fall in numbers acutely post-irradiation.

Macrophages make up the largest proportion of immune cells within the meninges, and dural macrophages can be subdivided into two populations delineated by their major histocompatibility complex class II (MHCII) expression (MHCII-high and MHCII-low) [30]. We next investigated whether macrophage phenotypes were altered in the dural meninges following RT to the abdomen. We observed a significant reduction in the proportion of MHCII-high macrophages in the dura (Fig. 2m), and the median fluorescent intensity (MFI) of macrophage MHCII expression was halved at day 7 post-RT (Fig. 2n). MHCII-high macrophage proportions and MFI of MHCII were restored by day 14 post-RT, correlating with resolution of RT-induced gut inflammation (Fig. 2m and n). Together, these data highlight the capacity for intestinal RT to robustly influence the immune landscape at the brain border tissues, altering both immune cell counts and macrophage phenotype.

### Gut-targeted RT induces immune population changes in the blood

Given that cells are able to translocate from the intestinal tissues to the dural meninges via the circulation, and the meningeal immune compartment is receptive to systemically derived signals, we next wanted to probe the RT-induced changes in the peripheral blood at days 7, 15, and 21 post-treatment (Fig. 3a; gating strategy, mean cell counts, and total CD45+ counts in Supplementary Figure S3a–c). Neutrophil and monocyte counts were significantly elevated in the blood at day 7 post-RT (Fig. 3b and c). However, there were significant reductions in eosinophil cell counts (Fig. 3d), which contrasted the local eosinophilia seen in the small intestine and colon at day 7 post-RT (Fig. 1i and q) but reflected the observed reduction in dural eosinophil numbers (Fig. 2f). At day 15 and 21, neutrophil populations remained persistently elevated, whilst monocytes and eosinophils returned to baseline numbers (Fig. 3b–d).

**Figure 3:**
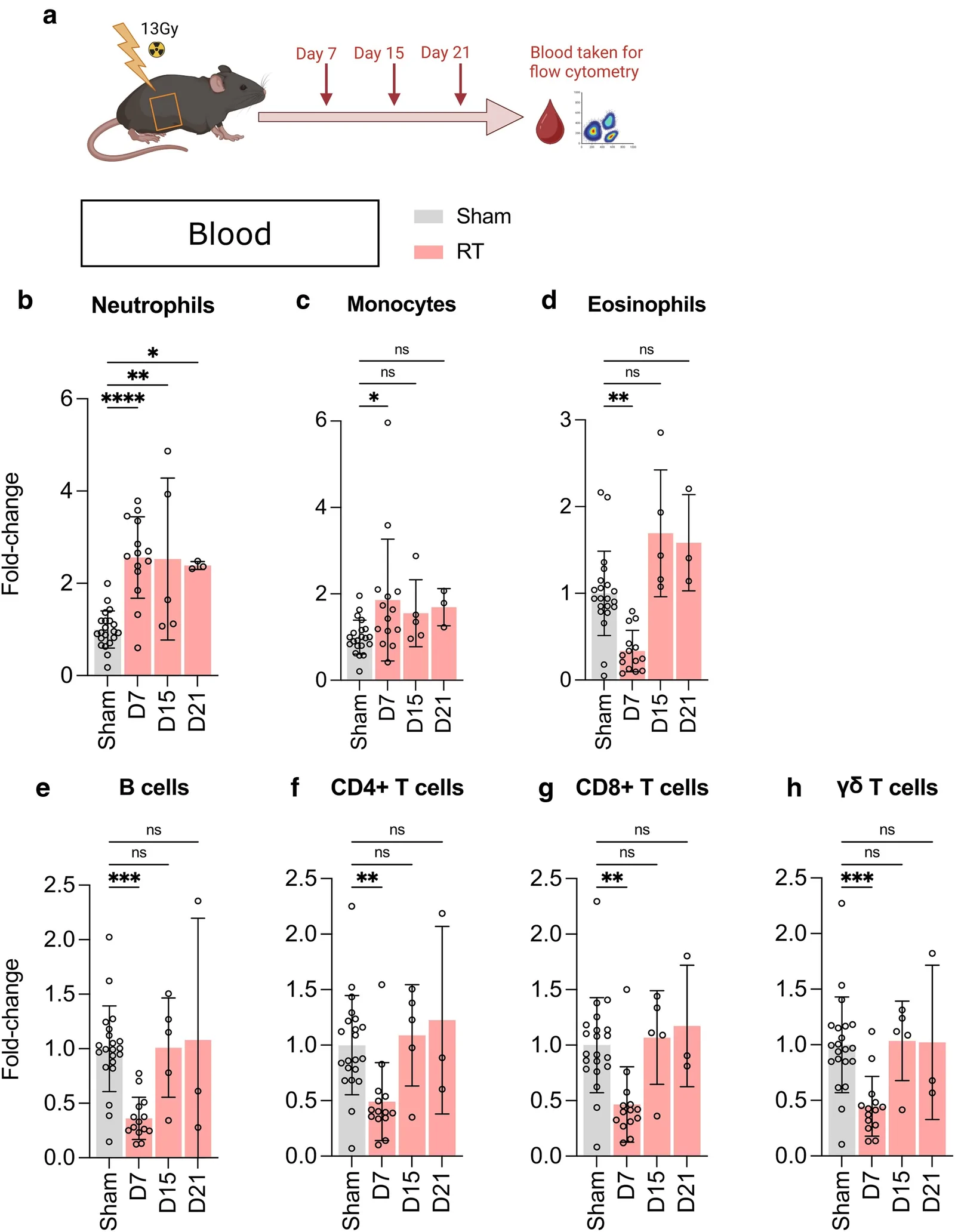
Immune cell changes in the peripheral blood following abdominal RT. (a) Mice received 13 Gy abdominal RT, and peripheral blood was collected for analysis by cardiac puncture at day 7 (D7), 15 (D15), and 21 (D21) post-treatment. (b-h) Flow cytometry time course analysis of changes in immune cell counts in the blood of sham vs irradiated mice. Data are presented as fold-change in cell counts per millilitre of blood normalized to experimental sham controls. All time points include at least *n* = 3 irradiated mice and shams across each time point were pooled. (b-h) Data analysed by one-way ANOVA with Dunnett’s post-hoc test or Kruskal–Wallis test with Dunn’s post-hoc test where appropriate. Data are presented as means ± SD. *P < 0.05 **P < 0.01 ***P < 0.001 ****P < 0.0001.

Circulating lymphocyte populations were also affected, marked by significant reductions in B cell, CD4+ T cell, CD8+ T cell, and γδ T cell counts at day 7 post-RT (Fig. 3e–h). These differences in the blood reflect changes seen in the dural meninges at the same timepoint (Fig. 2i–k) but were fully resolved by day 15 post-RT (Fig. 3e–h), aligning with resolution of local gut inflammation.

Together, these data demonstrate that gut-targeted RT triggers systemic responses in the peripheral blood affecting both myeloid and lymphocyte populations. These systemic immune cell changes largely reflect changes seen in the dural meningeal tissue, and normalized in line with resolution of gut inflammation, with the exception of neutrophils, which remained elevated for at least 3 weeks post-RT.

Given that the changes seen in the systemic blood mirror changes observed in dural immune populations, it was important to determine whether the dural changes could be due to contaminating blood within preps. To this end, we labelled blood leukocytes with an IV-injected CD45.2-FITC antibody prior to perfusion and analysed dural immune changes at day 7 following RT. We directly compared the fold-changes in immune cell counts of the whole dural meninges sample (total cells including meningeal and any contaminating blood cells) and CD45.2-FITC negative (CD45.2 -ve) immune cells, comprising only meningeal cells following gating to remove any contaminating CD45.2 antibody-labelled blood cells (example gating in Supplementary Figure S4a). We saw no difference in the extent of RT-induced immune modulations in CD45.2-negative dural immune cells (Supplementary Figure S4b), confirming that gut RT is directly affecting immune populations within the dural meninges.

### Depletion of the gut microbiota with broad-spectrum antibiotics partially reduces local intestinal inflammatory responses to RT

The gut microbiota is a key player in mucosal immunity and influences intestinal inflammatory responses. As intestinal RT causes significant inflammation and a breakdown of the mucosal barrier (Fig. 1) [10], this could potentially lead to the translocation of luminal bacteria that contribute to inflammatory signals within the intestine. To probe how the gut microbiota might be influencing both the local and systemic immune responses to abdominal RT, we treated mice with a cocktail of broad-spectrum antibiotics (Abx) to deplete the gut microbiota as previously described [27], for 2 weeks prior to RT and continuously until the end of the experiment (Fig. 4a). Treatment with Abx to deplete the gut microbiota modulated the dynamics of weight change across the experiment without altering peak weight loss (Fig. 4b).

**Figure 4:**
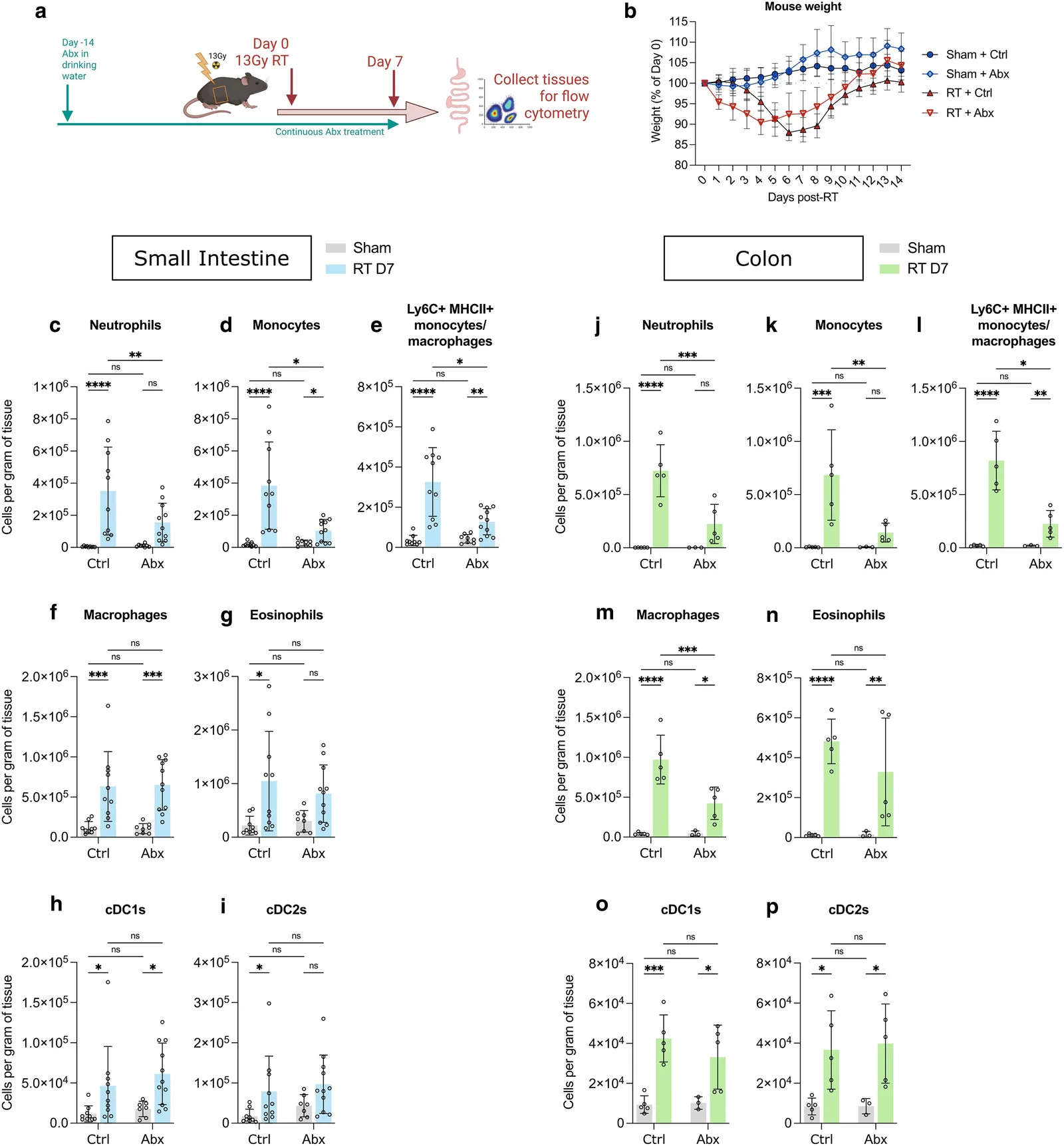
Effect of antibiotic treatment on the local inflammatory response to abdominal RT. (a) Mice were given either Abx to deplete the gut microbiota or Ctrl before receiving 13 Gy or sham abdominal irradiation. Gut tissues were collected for flow cytometry analysis at day 7 post-treatment. (b) Percentage body weight loss in Abx- and Ctrl-treated, irradiated vs sham mice between days 0 and 14 following treatment. (c-p) Flow cytometry analysis of changes in immune cell counts in the small intestine (c-i) and colon (j-p) of sham vs irradiated mice following Abx or Ctrl treatment at day 7 post-RT (small intestine *n* = 8–11 per group from two experimental repeats; colon *n* = 3–5 per group). (c-p) Data analysed by two-way ANOVA with Fisher’s LSD post-hoc test or ART analysis with ANOVA where appropriate. Data are presented as means ± SD. *P < 0.05 **P < 0.01 ***P < 0.001 ****P < 0.0001.

To explore whether Abx treatment was affecting local inflammatory responses in the intestine, the small intestine and colon tissues were analysed by flow cytometry at day 7 post-RT. In line with previous descriptions (Fig. 1d–s), irradiation of Ctrl-treated animals induced a significant myeloid-driven response in both the small intestine and colon, marked by increases in neutrophil, monocyte, macrophage, eosinophil, and dendritic cell populations (Fig. 4c–p). However, when mice were pretreated with Abx, the extent of myeloid immune infiltration following RT was partially stunted compared to Ctrl-treated animals. Specifically, in the small intestine, cell counts for neutrophils, monocytes, and Ly6C+ MHCII+ transitioning monocytes/macrophages were significantly lower in Abx-treated mice compared to Ctrl-treated mice following RT (Fig. 4c–e). There were no significant differences in macrophage, eosinophil, cDC1, and cDC2 counts between Abx- and Ctrl-treated mice receiving RT (Fig. 4f–i). However, Abx-treated mice no longer showed statistically significant increases in eosinophil and cDC2 cell counts in sham vs irradiated animals (Fig. 4g and i). Similar to the small intestine, in the colon, cell counts for neutrophils, monocytes, and Ly6C+ MHCII+ transitioning monocytes/macrophages, as well as macrophages, were significantly lower in Abx-treated mice compared to Ctrl-treated mice following RT (Fig. 4j–m). However, Abx treatment had no impact on RT-induced increases in colonic eosinophil, cDC1, and cDC2 cell counts (Fig. 4n–p).

Together, these data highlight a role for the microbiota in promoting RT-induced inflammation in the small intestine and colon, likely due to biota-derived pathogen-associated molecular patterns (PAMPs) exacerbating inflammation following gut barrier breakdown.

### Depletion of the gut microbiota with broad-spectrum antibiotics reduces RT-induced systemic immune changes in the dural meninges and peripheral blood

In addition to mediating local intestinal immune responses, the gut microbiota can influence immune responses across systemic tissues including the meninges [25, 28, 31]. We therefore next investigated whether the gut microbiota plays a role in mediating the systemic immune changes seen in the dural meninges and blood after gut-targeted RT. Mice were given Abx or Ctrl treatment, and the dural meninges and blood were analysed at days 7 and 15 post-abdominal RT (Fig. 5a).

**Figure 5:**
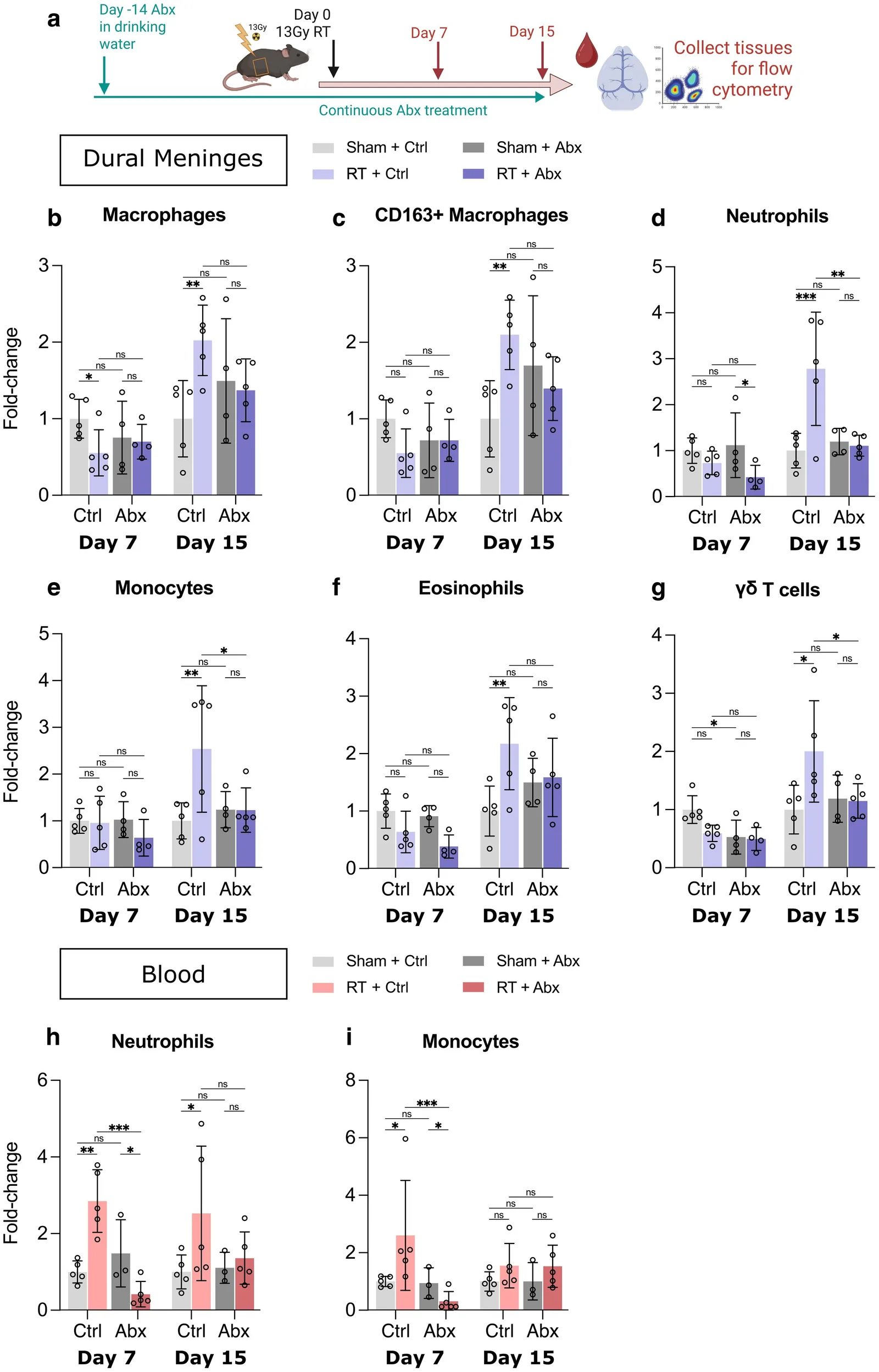
Effect of antibiotic treatment on the systemic inflammatory responses to abdominal RT. (a) Mice were given either Abx to deplete the gut microbiota or Ctrl before receiving 13 Gy or sham abdominal irradiation. The dural meninges and blood were collected for flow cytometry analysis at day 7 and day 15 post-treatment. (b-i) Flow cytometry analysis of fold-changes in immune cell counts in the dural meninges (b-g) and blood (h, i) of sham vs irradiated mice following Abx or Ctrl treatment (dura *n* = 4–5 per group; blood *n* = 2–5 per group). (b-i) Data analysed by two-way ANOVA with Fisher’s LSD post-hoc test or ART analysis with ANOVA where appropriate. Data are presented as means ± SD. *P < 0.05 **P < 0.01 ***P < 0.001.

Analysis of the meningeal tissue showed that RT-induced immune changes could be modulated by Abx treatment. Thus, Abx treatment reversed the RT-induced reduction in dural macrophages at day 7 post-RT (Fig. 5b), as well as the RT-induced increases in dural macrophages (both total and CD163+ BAM), neutrophils, monocytes, eosinophils, and γδ T cells at day 15 post-RT (Fig. 5b–g). In contrast, significant reductions in B cell counts following RT at day 7 were not affected by Abx treatment, although the enhancement in B cells seen at day 15 post-RT was partially reversed (Supplementary Figure S5a). Abx treatment did not affect the other dural immune cells measured (Supplementary Figure S5b–e). Together, this implicates the gut microbiota in mediating some, but not all, systemic immunomodulatory effects seen in the dural meninges, most prominently affecting the myeloid immune cell types at the day 15 timepoint.

In the blood, influences from the microbiota were most prominent in neutrophil and monocyte populations. Depletion of the gut microbiota with Abx treatment ameliorated the RT-induced elevations in neutrophils observed at days 7 and 15 post-RT (Fig. 5h), as well as the elevation in monocytes seen at day 7 post-RT (Fig. 5i). However, RT-induced reductions in eosinophils and B cells appeared to be unaffected by depletion of the microbiota in conjunction with RT treatment (Supplementary Figure S5f and g). Antibiotic treatment did not affect the other circulatory immune cells measured (Supplementary Figure S5h–j).

Collectively, in both systemic tissues analysed, the presence of an intact gut microbiota had the strongest effect on RT-induced alterations in neutrophil and monocyte populations. In the blood, this effect was seen most prominently at day 7, aligning with Abx-ameliorated myeloid responses in the intestinal tissues. However, in the dura, Abx treatment most strongly impacted RT-induced immune changes at day 15, suggesting a delay in the microbiota-mediated systemic responses in the dural meninges. Together, these data highlight a role for the gut microbiota in exacerbating the local gut inflammation and having downstream influences on systemic immune responses in the blood and brain borders.

### Gut-targeted RT induces anxiety-like phenotypes in mice

We next investigated whether gut-targeted RT had any consequences on behaviour as the meningeal immune system can signal to the brain in a manner that can modulate behaviour in mice [17, 32–35]. To test this, we carried out a series of behavioural tests including OF, EPM, and NOR to assess for anxiety-like behaviours and short-term memory deficits. Mice were treated with 13 Gy RT and allowed to recover before carrying out behavioural testing between days 14 and 16 post-RT (Fig. 6a). Data from the OF and NOR tests confirmed that there was no residual sickness behaviour from the RT treatment at this timepoint as there was no difference in total distance travelled in OF or in total time spent investigating both objects combined in NOR by RT-treated mice compared to sham controls (Fig. 6b and c). The OF test showed no differences in time spent in the inner zone of the arena between sham and RT mice (Fig. 6d). However, mice that received RT treatment had fewer entries into the open arms of the EPM and spent less time exploring the open arms of the maze compared to sham animals, indicating anxiety-like behaviours that can be observed following recovery of the mice post-abdominal RT (Fig. 6e). There were no observed cognitive effects, as demonstrated by appropriate increased exploration of the novel object in the NOR test by RT-treated mice, and no differences in the object discrimination index between the RT and sham groups (Fig. 6f). Overall, this suggests that RT targeted to the gut can have systemic consequences affecting anxiety-like behaviour in mice but there are no short-term memory deficits at day 15 post-irradiation. These anxiety-like changes occur at resolution of intestinal inflammation but coincide with dural immune changes at the same point, though the direct contribution of meningeal immune populations to behaviour in this context is yet to be tested.

**Figure 6:**
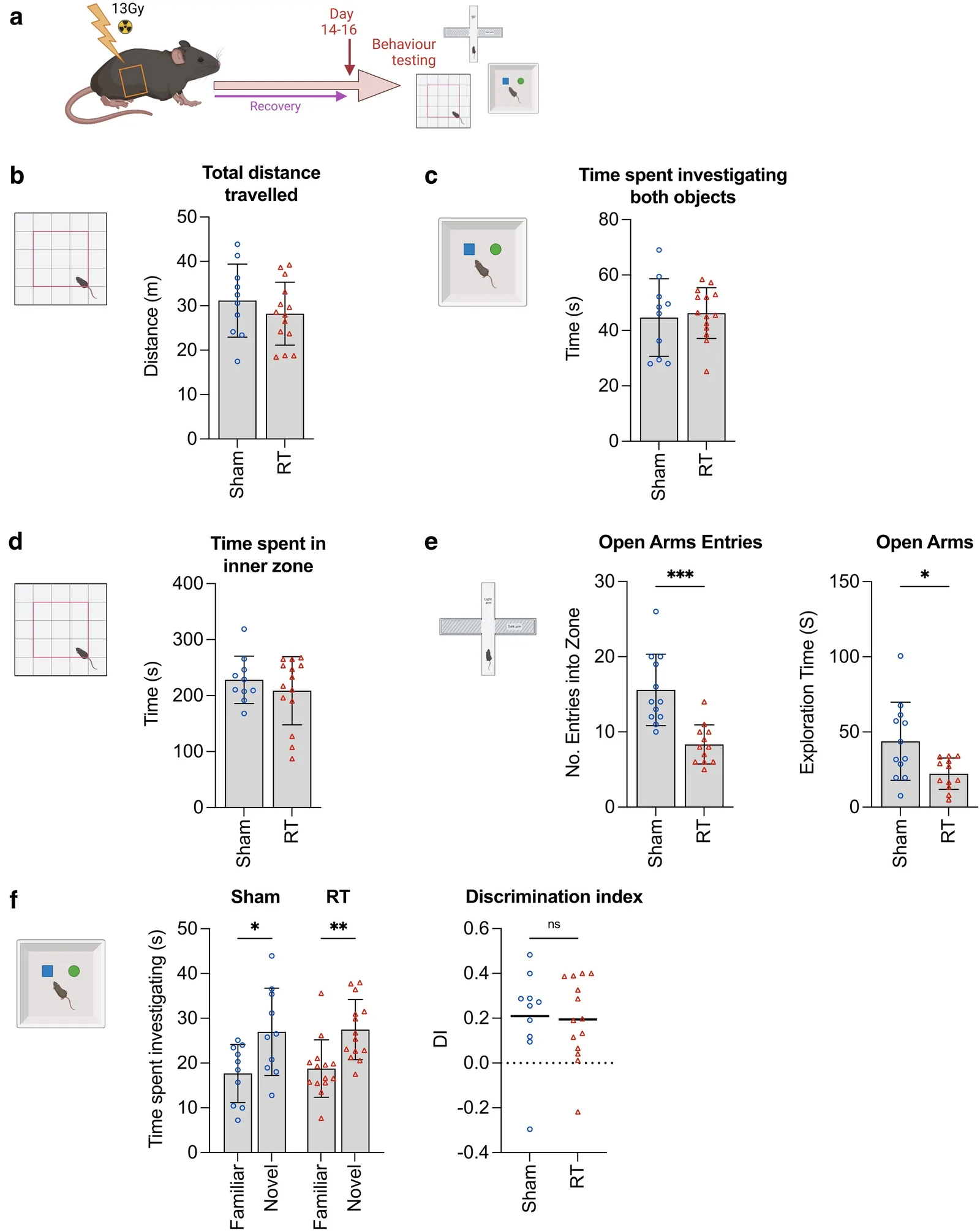
Effect of abdominal RT on behaviour. (a) Mice were given 13 Gy abdominal irradiation and allowed to recover before being subjected to behavioural testing between days 14 and 16 post-RT. Distance travelled in the OF maze (b) and combined time spent investigating both objects in the NOR test (c) acted as a surrogate for overall wellbeing. Tests for anxiety-like behaviours and cognitive defects included OF (d), EPM (e) and NOR (f). *n* = 10–14 per group. (b-f) Data analysed by unpaired *t*-test or Mann–Whitney test where appropriate. Data are presented as means ± SD. *P < 0.05 **P < 0.01 ***P < 0.001.

## Discussion

The importance of immune organ crosstalk is increasingly recognized [6], but the local and systemic RT-induced immune responses are poorly understood. Here we show that RT targeted to the intestine induces systemic immunomodulatory effects in the dural meninges that the gut microbiota plays an important role in driving these systemic responses, and that abdominal RT treatment induces anxiety-like behaviours in mice. Irradiation of the small intestine and proximal colon induces tissue damage and inflammation in the local tissues, predominantly through myeloid cell recruitment. In-depth analysis of these locally expanded populations has been described previously [10, 36], identifying multiple macrophage clusters following RT treatment, that present with pro-inflammatory and repair-associated phenotypes. The gut microbiota is important in driving this immune response, likely due to infiltration of luminal bacterial products into the lamina propria following RT-induced gut barrier breakdown. Antibiotics given before abdominal radiation in mice are known to reduce lipopolysaccharide (LPS) content in the ileum after radiation, and antibiotic-treated mice have improved survival and reduced expression of inflammatory markers in intestinal tissue [37]. This is in line with our observations that antibiotic treatment can dampen local inflammation in the gut following RT. However, the RT-induced local inflammatory response is not totally ameliorated, likely due to the presence of DAMPs which still drive recruitment of myeloid immune cells following RT-induced tissue damage even in the absence of an intact microbiota [38].

Here we modelled intestinal RT with a single 13 Gy abdominal dose as previously described [10]. Clinically, abdominal and pelvic RT is typically delivered as 40–50 Gy doses, administered in 1.8–2 Gy daily fractions over several weeks [39]. Although this model does not directly replicate the fractionated RT regimes used clinically, it induces intestinal injury that recapitulates many of the GI toxicities observed in patients. Furthermore, while this model does not incorporate the presence of a tumour, portions of the bowel are frequently exposed during abdominal RT, and GI toxicity remains a common treatment-related complication. Therefore, this model provides a useful platform for investigating how local radiation-induced intestinal injury can drive systemic effects beyond the irradiated tissue.

Beyond the intestinal tissues, abdominal RT drives systemic changes in the peripheral blood. RT-induced neutrophil and monocyte expansion in the blood were microbiota-driven, whilst systemic reductions in eosinophils and B cells were not impacted by depletion of the gut microbiota. When the gut becomes inflamed, cytokine and chemokine signals stimulate haematopoietic stem cell proliferation and differentiation in the bone marrow, mobilization of immune cells to the peripheral blood, and homing to the inflamed gut [40]. In our model, most blood immune alterations peaked and resolved in conjunction with resolution of intestinal inflammation, suggesting a close link between systemic blood responses and the gut, although neutrophilia persisted beyond day 21. Treatment with antibiotics prior to RT ameliorated RT-induced blood neutrophil and monocyte expansion, as similarly seen in the small intestine and colon. It has been shown that depletion of the gut microbiota with antibiotics can impair bone marrow haematopoiesis marked by reduced bone marrow progenitor proliferation and a reduction in both myeloid- and lymphoid-associated multipotent progenitors [41]. In conjunction with reduced PAMP signals in the gut, impairment in bone marrow haematopoiesis could contribute to the amelioration of neutrophil and monocyte expansion in the blood and intestinal tissues of mice treated with antibiotics alongside RT.

During peak intestinal inflammation, dural immune changes largely reflect those observed in the peripheral blood, however, alterations persist beyond days 14 and 21, suggesting sustained immune remodelling in the dural meninges even after systemic inflammation begins to resolve. Immune cells have the capacity to migrate from the intestines to the dura mater. Photoconverted cells from the gut can be detected in the meninges in both homeostasis and inflammation, and the dura mater has been shown to home gut-educated IgA plasma cells [23, 25]. In a DSS colitis model, dural IgA plasma cells and B cells were seen to increase in number during resolution of intestinal inflammation, supporting our observed elevations in B cells at day 14/15 post-RT. It has been shown that these responses are important to protect the brain from spread of potential intravenous pathogens, as depletion of these dural IgA plasma cells resulted in increased fungal load in the brain and increased mortality following systemic injection of *Candida albicans* [23]. This suggests that the expansion of dural immune cells we see in our model at day 14 could be part of a similar protective response. However, prior to this expansion, we see a significant reduction in B cells, as well as T cells, eosinophils, macrophages, and dendritic cells. Furthermore, we see a reduction in the antigen-presentation capacity of dural macrophages, marked by reduced MHCII expression. This change in MHCII expression on dural macrophages has been similarly observed in models of systemic lymphocytic choriomeningitis virus (LCMV) infection and peripheral LPS injection [22].

Dural macrophages are known to present CNS-derived self-antigen to lymphocytes to promote local tolerance and maintain homeostasis within the brain border tissues [42]. The reduction in MHCII expression that we observe may represent a protective mechanism that limits adaptive immune activation and subsequent reactivity to CNS antigens in inflamed contexts. This is seen more broadly in severe brain injuries such as stroke, which can result in peripheral immunosuppression [43, 44]. This may protect the brain from autoimmune mechanisms; however, it can also leave it more susceptible to infection. It has been shown that depletion of MHCII-high meningeal macrophages results in increased penetration of LCMV into the brain when infected peripherally, highlighting the importance of these meningeal immune populations in protecting the CNS [22]. Following RT, we first see a reduction in immune cell counts followed by significant increases compared to sham animals. This could suggest a temporal shift between an initial immune-suppressive state during the peak of inflammation, followed by a resurgence in immune cell counts to provide protection from pathogens that might enter the circulation. Further time course analyses in additional gut and systemic inflammatory models, including analysis of the local signals mediating these pro/anti-inflammatory shifts, would be required to better understand the meningeal immune response to gut irradiation.

We found that depletion of the gut microbiota prevented RT-induced elevations in neutrophil, monocyte, macrophage, eosinophil, and γδ T cell populations in the dural meninges at day 15 post-RT. This supports other work that shows gut microbiota can influence meningeal immune cell types. For example, homeostatic dural IgA+ plasma cells are lost in antibiotic-treated mice [23], migration of gut-derived NK cells to the dura is reduced in germ-free and antibiotic-treated mice [25], and antibiotic treatment increases dural regulatory T cell (Treg) expansion and reduces IL-17+ γδ T cell populations in the context of experimental stroke [29]. Our data suggest that myeloid, but not lymphoid, cell changes are gut microbiota-dependent after intestinal RT. Further study would be required to explore what delineates the microbiota-responsive and microbiota-non-responsive populations in the dural meninges following gut radiation and whether changes are driven by direct microbial signals or by downstream effects of reduced inflammatory signals originating from the gut and circulation.

Meningeal immune populations are maintained through local self-renewal of long-lived resident cells, recruitment from the peripheral circulation, and, uniquely, direct migration of immune cells from the calvarial bone marrow via specialized channels [45–48]. Although gut inflammation has been proposed to promote immune cell trafficking from the intestine to the dura mater [23, 24], this alone may not explain the dynamic immune changes observed throughout our time course post-RT. Brain pathology can stimulate calvarial haematopoiesis and subsequent immune cell migration into the dura and brain [47], but whether the calvarial bone marrow also responds to peripheral inflammatory or microbiota-derived signals remains unknown. This could possibly contribute to the microbiota-dependent dural immune alterations observed following abdominal RT.

To probe the possible brain functional consequences of RT-induced intestinal inflammation, we looked at the behaviours of mice following treatment. Sham and irradiated mice spent equal time in the inner zone of the OF test but showed anxiety-like phenotypes in the more sensitive EPM. Short-term memory was found to be intact in RT-treated mice, however, another study showed that mice receiving 10 Gy total abdominal irradiation performed worse in the Morris water-maze test, suggesting an impairment in spatial learning and memory post-RT [49]. Furthermore, a previous study in rats showed no differences in behavioural test performances between abdominally irradiated animals vs control, albeit the radiation dose was over half that of our model and therefore would have elicited reduced inflammatory effects [50]. It is understood that patients can experience psychosocial decline throughout treatment [1, 3, 4], however, given the multitude of factors that could be contributing to a patient’s psychological state following a cancer diagnosis and side effects of anti-cancer therapy, it is difficult to establish causation. It must also be noted that transfer of luminal bacterial products, such as LPS, to the blood, as mentioned above, could directly inflame the blood-brain barrier (BBB) and cause BBB breakdown with consequences for brain function [51–53]. Although behavioural testing in the open field and NOR test showed no overt sickness behaviour after intestinal RT, changes in exploration or deficits in cognitive function are observed in models that administer exogenous LPS [51–53]. Thus, it is possible that chronic low-level exposure of LPS to the BBB after intestinal RT could contribute to the subtle anxiety-like behaviours we observe in our models, similar to the effects seen in mice fed high-fat diets [54]. Further in-depth assessment of the BBB in conjunction with the dural meninges over more extended time points in a variety of tests would be required to definitively conclude how the local and systemic healthy tissue toxicities associated with pelvic RT could be having an impact on the systemic immune response and how that might affect the psychosocial state of cancer patients.

Further extensions to the current work would be to investigate sex-dependent differences in the immune response. Here, we selected female mice to minimize variability arising from known sex-dependent differences in intestinal radiosensitivity and dural immune cell compositions [55, 56]. Future studies should include both sexes to establish how sex, RT, and site-specific immune responses interact. In addition, although we provide strong evidence of gut-targeted RT driving systemic immunomodulatory effects in the dural meninges, whether and how these meningeal immune responses influence the brain has not been formally tested. Additional understanding of the effects of RT on the blood-brain barrier and blood-cerebrospinal fluid barrier will be important for determining whether the behavioural changes observed following RT are mediated by meningeal immune alterations, as has been reported in other contexts [17, 32–35, 57, 58].

In conclusion, abdominal irradiation, which incurs local tissue damage and inflammation in the small intestine and colon, can systemically impact immune cell populations residing in the brain borders. Depletion of the gut microbiota with broad-spectrum antibiotics was able to reduce some of the observed RT-induced dural immune changes. This demonstrates that the dose-limiting radiation injury that is affecting healthy intestinal tissue in pelvic RT treatment is having consequences beyond local inflammation that reach the brain borders and may affect both immune defence and homeostatic function. Together, our results show that abdominal RT leads to systemic immunomodulatory and functional effects within the CNS, highlighting the need to further understand the underlying immunological mechanisms in developing therapeutic interventions to further improve cancer outcomes.

## Supplementary Material

kyag019_Supplementary_Data

## Data Availability

The data underlying this article will be shared on reasonable request to the corresponding author.

## Abbreviations:

Abx: antibiotics
ART: aligned rank transform
BAM: border-associated macrophage
BBB: blood-brain barrier
CBCT: cone-beam computed tomography
cDC1: conventional type 1 dendritic cell
cDC2: conventional type 2 dendritic cell
CNS: central nervous system
Ctrl: control
DAMP: damage-associated molecular pattern
DI: discrimination index
DSS: dextran sodium sulphate
EPM: elevated plus maze
GI: gastrointestinal
H&E: haematoxylin and eosin
IV: intravenous
LCMV: lymphocytic choriomeningitis virus
LPS: lipopolysaccharide
LSD: least significant difference
MFI: median fluorescence intensity
MHCII: major histocompatibility complex class II
NK: natural killer
NOR: novel object recognition
OF: open field
PAMP: pathogen-associated molecular pattern
RIII: radiation-induced intestinal injury
RT: radiotherapy
SARRP: Small Animal Radiation Research Platform
SD: standard deviation
TCR: T-cell receptor
Treg: regulatory T cell
